# The Diagnostic Accuracy and Clinimetric Properties of Screening Instruments to Identify Frail Older Adults Attending Emergency Departments: A Protocol for a Mixed Methods Systematic Review and Meta-Analysis

**DOI:** 10.3390/ijerph19031380

**Published:** 2022-01-26

**Authors:** Elizabeth Moloney, Duygu Sezgin, Mark O’Donovan, Kadjo Yves Cedric Adja, Keith McGrath, Aaron Liew, Jacopo Lenzi, Davide Gori, Kieran O’Connor, David William Molloy, Evelyn Flanagan, Darren McLoughlin, Maria Pia Fantini, Suzanne Timmons, Rónán O’Caoimh

**Affiliations:** 1 Health Research Board Clinical Research Facility, Mercy University Hospital, T12 WE28 Cork, Ireland; markodonovan@ucc.ie (M.O.); e.flanagan@ucc.ie (E.F.); rocaoimh@hotmail.com (R.O.); 2 Department of Geriatric Medicine, Mercy University Hospital, T12 WE28 Cork, Ireland; kmcgrath@muh.ie (K.M.); koconnor@muh.ie (K.O.); w.molloy@ucc.ie (D.W.M.); 3 School of Nursing and Midwifery, College of Medicine, Nursing and Health Sciences, National University of Ireland, H92 N2R2 Galway, Ireland; duygu.sezgin@nuigalway.ie; 4 Department of Biomedical & Neuromotor Sciences, University of Bologna, 40126 Bologna, Italy; adjayvescedric@gmail.com (K.Y.C.A.); jacopo.lenzi2@unibo.it (J.L.); dedegori27@gmail.com (D.G.); mariapia.fantini@unibo.it (M.P.F.); 5 Clinical Sciences Institute, National University of Ireland, H91 TK33 Galway, Ireland; aaron.liew@nuigalway.ie; 6 Department of Endocrinology, Portiuncula University Hospital, H53 T971 Galway, Ireland; 7 Centre for Gerontology and Rehabilitation, School of Medicine, University College Cork, T12 WE28 Cork, Ireland; s.timmons@ucc.ie; 8 Department of Emergency Medicine, Mercy University Hospital, T12 WE28 Cork, Ireland; dmcloughlin@muh.ie

**Keywords:** frailty, frailty screening tools, emergency department, older adult, systematic review, diagnostic accuracy

## Abstract

Background: Prompt and efficient identification and stratification of patients who are frail is important, as this cohort are at high risk of adverse healthcare outcomes. Numerous frailty screening tools have been developed to support their identification across different settings, yet relatively few have emerged for use in emergency departments (EDs). This protocol provides details for a systematic review aiming to synthesize the accumulated evidence regarding the diagnostic accuracy and clinimetric properties of frailty screening instruments to identify frail older adults in EDs. Methods: Six electronic databases will be searched from January 2000 to March 2021. Eligible studies will include adults aged ≥60 years screened in EDs with any available screening instrument to identify frailty (even if not originally designed for this purpose). Studies, including case-control, longitudinal, and cohort studies, will be included, where instruments are compared to a reference standard to explore diagnostic accuracy. Predictive accuracy for a selection of outcomes, including mortality, institutionalization, and readmission, will be assessed. Clinical and methodological heterogeneity will be examined, and a random effects meta-analysis performed if appropriate. Conclusion: Understanding whether frailty screening on presentation to EDs is accurate in identifying frailty, and predicting these outcomes is important for decision-making and targeting appropriate management.

## 1. Introduction

Population ageing is challenging health-care systems worldwide. Between 2015 and 2050, the world’s population of older people is projected to triple, resulting in more older adults living longer with chronic conditions [1,2]. This pressure is being particularly felt by hospital emergency departments (EDs), which are seeing increasing numbers of patients aged over 65 years presenting acutely unwell [3]. Frailty, an age-associated risk state predisposing individuals to a range of adverse outcomes [4], is one of the most important global problems associated with an ageing population and is increasingly recognized as an emerging public health concern [5]. Though frailty is common in community-dwelling older adults [6], it is also highly prevalent across a range of healthcare settings [7], including amongst those attending EDs [8]. Frailty, though it has no single widely accepted definition, is associated with an increased incidence of functional decline, falls, delirium, and disability [4].

Older adults account for up to a quarter of all ED visits [9]. These patients have a distinct pattern of care needs [10]. Providing a goal-directed model of care, a focus on what matters most to patients based on their stated preferences to guide their treatment decisions [11], can be particularly challenging to deliver in this rapid, fast-paced environment. Compared to younger patients, older patients with frailty experience more adverse outcomes after attending EDs, namely longer length of stay [12] and higher inpatient mortality [13]. Identifying frail older adults is challenging. Frailty is a complex process that can be overlooked given its slowly progressive nature [14]. It can be difficult to separate from acute illness [15]. Early recognition of frailty in EDs can reduce the incidence of adverse events; aid discharge planning [16]; direct patients to the most appropriate care setting [17]; alert specialist ‘frailty intervention therapy’ (FIT) teams to undertake comprehensive geriatric assessment (CGA) [17]; aid in targeted treatment planning, including medication review [18], advanced care planning, and end-of-life care for severely frail patients [19] and alert specialist ‘frailty intervention therapy’ (FIT) teams to undertake comprehensive geriatric assessment (CGA) [20]. Increasing evidence from international studies supports the introduction of these FIT teams of health and social care professionals to the ED to provide specialized interdisciplinary care. These typically include physiotherapists, occupational therapists, and medical social workers, which provide early assessment and intervention to older adults in the ED and can lead to safer discharges and increased patient and staff satisfaction [20].

Support for early risk stratification in EDs is growing, with the recent launch of European Geriatric Emergency Medicine (GEMS) Guidelines at the 17th European Geriatric Medicine Society (EUGMS) congress in Athens, Greece. Included among the recommended guidelines was utilization of age/frailty risk stratification using frailty screening tools in EDs [21]. At present, several barriers prevent frailty from being rapidly recognized in the ED. The most important of these is that methods to identify frailty are overly complicated for use in EDs and are usually completed after patient treatment decisions have been enacted [22]. There are two broadly accepted methods to measure frailty: the Fried phenotype approach and the Rockwood cumulative deficit approach. Both frailty models can be used to predict increased risk of prolonged hospitalization, institutionalization, and inpatient mortality [23]. However, both can be difficult to apply in an ED setting. The Fried phenotype, focusing on physical signs and symptoms of frailty, requires tests and information that are not readily available or impractical to perform in EDs [23]. The cumulative deficit model uses a count of items or “deficits” across physical, cognitive, mental health, and functional domains to create an index using variables based on a CGA [24], but is complex, potentially time-consuming, and also difficult to operationalize in the ED [25]. In addition, a number of frailty-oriented risk-stratification tools that were not designed to measure frailty have been developed or redeployed to identify increased risk of adverse outcomes among older adults, including amongst those who are frail [26,27]. One of the most widely used instruments in ED is the Identification of Seniors At Risk (ISAR) screening tool [28]. Such instruments are quick to administer; include variables associated with poor healthcare outcomes; and are validated to predict future risk of adverse events, including mortality, hospital readmission, institutionalization, and more recently, frailty [29]. Hence, there are a wide variety of instruments and approaches available to identify vulnerable older people in EDs. All differ in terms of clinimetric properties (reliability, sensitivity, specificity, validity, etc.) [30], diagnostic accuracy for frailty and other outcomes, expertise required, and the time needed for application. However, to date, few studies have been published examining and comparing these instruments in EDs, which is important, as to our knowledge none of these were originally designed for use in this setting.

Indeed, the ED is a very specific setting where there is a need for efficient (time sensitive, “short”, and easy to score), yet accurate (to minimize false negatives), instruments. A systematic review by Elliott et al. (2017) focused on the feasibility of implementing risk stratification tools for older persons in EDs [30], suggesting the need for further work to be undertaken to understand risk-stratification tools in urgent care settings. A previous umbrella review of short frailty screens by Apostolo et al. (2017), with a literature review up until 2015, was not ED-specific and did not perform a meta-analysis [31]. Since that systematic review, the frailty identification and management of frailty in ED has become more important in the face of COVID-19. The ED is a specific setting with defined challenges. Rapid frailty triage screening and escalation of care decisions have played an important role in resource allocation during this pandemic [32]. It is an optimum time to undertake this systematic review in order to address the recommendations from previous studies, and evaluate whether instruments that are currently being used to briefly identify frailty and prognostics in this setting are accurate, and, if so, which is the optimal choice.

Although most available scales perform better than chance in predicting a range of poor outcomes, they have variable performance in the community [33], medical assessment units [34], and EDs [35]. In 2014, another review found that few risk-prediction instruments were available to screen older adults for risk of adverse outcomes in EDs [36], highlighting the need to evaluate existing short risk-prediction instruments for risk of adverse events among older adults attending EDs, and to compare their clinimetric properties with established short screening instruments for frailty. If progress is to be made to manage the care needs of increasing numbers of complex and frail older patients presenting to EDs, then recognition and response systems should be introduced as an imperative. Understanding which short screening instruments, either frailty-specific or non-specific, are most accurate at identifying older patients who may experience adverse outcomes, or those who require tailored medical management of peri-operative optimization, is a central component of such systems. Despite this, insufficient attention has been paid to the clinical utility of such tools, including ease of use and the level of training required to be able to complete them. An instrument can have excellent reliability and validity, but these benefits will not be realized if the instrument is too cumbersome for staff to use, takes too long, or can only be used by a few trained people.

Study protocols are an integral part of medical research and should always be made available in the public domain [37]. Many groups have called for the widespread preparation and registration of systematic review protocols in order to increase the availability and accessibility of a priori methods for systematic reviews [37]. This study protocol follows in the steps of FRAILTOOLS [38] and Higginbotham et al. [39] in providing a clear, transparent roadmap for our systematic review. Given the multitude of frailty instruments now published, this protocol may reduce duplication of efforts by other research teams by providing explicit documentation of our research plan and establishes our peer reviewed roadmap.

Further, given the importance of, and challenges associated with, identifying frailty in EDs, the systematic review and meta-analysis described in this study protocol aims to: (1) review the evidence regarding the diagnostic accuracy to identify frailty and subsequent risk of adverse outcomes, and (2) to evaluate the clinimetric properties of current frailty screening tools and brief frailty-oriented risk-stratification instruments used in EDs for older people.

## 2. Materials and Methods

### 2.1. Study Design and Registration

**Figure 1:**
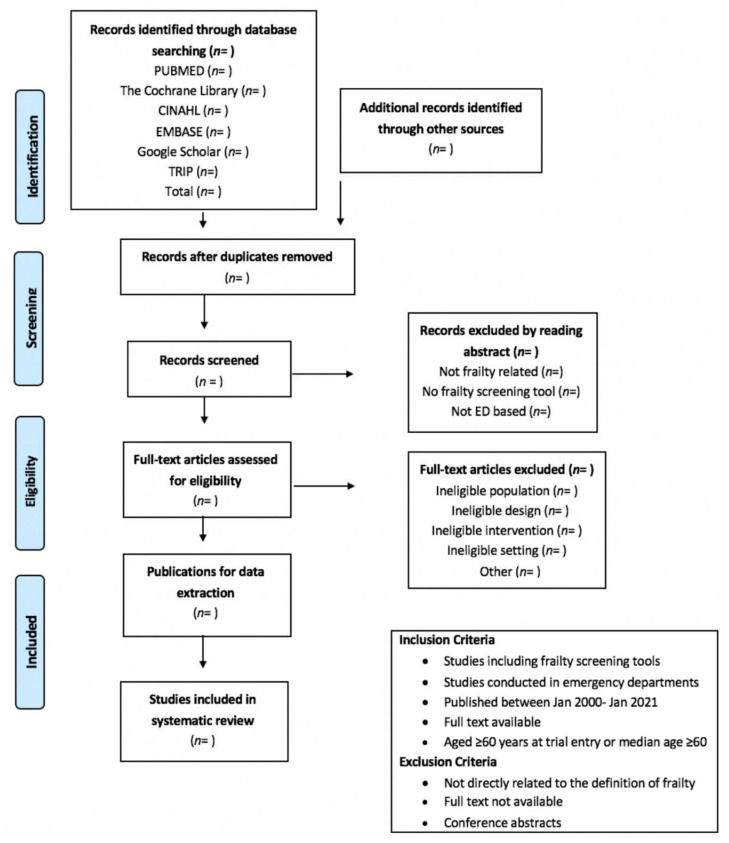
Example of the PRISMA flow diagram to be used in this study.

This systematic review and, if applicable, meta-analysis, will identify studies that have reported on validated screening instruments among older adults in ED settings, examining the properties of those used to identify older adults as frail, including those not designed originally to measure frailty, but which are used for this purpose, e.g., short risk-prediction instruments [27]. The review will conform to the principles outlined in the Cochrane Handbook for Systematic Reviews of Diagnostic Test Accuracy, and the Preferred Reporting Items for Systematic Reviews and Meta-Analyses Protocols (PRISMA) standardized reporting guidelines will be referenced. The Preferred Reporting Items for Systematic Reviews and Meta-Analyses Protocols (PRISMA-P) guidelines will be used in completing this review protocol [40]. An example of the PRISMA flow diagram to be used with this study is provided in Figure 1.

The United Nations definition of an older person includes those aged over 60 years of age [41]. The World Health Organization and the ATHLOS research project on ageing emphasize that many social and political factors influence ageing, i.e., that individuals do not all become “old” at the same age [42]. A number of factors, including nutrition, migration, displacement, and inequities in access to healthcare, often adversely affect less-developed nations, such that these populations effectively “age” faster. Hence, for the purposes of this study protocol, adults aged ≥60 years are included to reflect the best global practice among these international organizations who advocate for older adults.

The protocol is registered on the International Prospective Register of Systematic Reviews (PROSPERO), trial registration number CRD42020216780. By requiring the documentation of a priori methods, this register facilitates increased transparency in the review process by allowing readers of systematic reviews to compare methods, outcomes, and analyses carried out with those planned in advance, and judge whether such changes impact the results of a review [40].

### 2.2. Eligibility Criteria

As this is a mixed methods review, question formats will be tailored to the review type [43]. The taxonomy and definitions that will be used for the clinimetric properties evaluated will follow criteria established by the COnsensus-based Standards for Health Measurement INstruments (COSMIN) [38,44], and are:

- Validity: refers to the extent to which an instrument measures the construct(s) for which it was constructed, including: content validity, construct validity, and criterion validity (concurrent validity, predictive validity).
- Reliability: highlights elements related to coherence, accuracy, stability, equivalence, and homogeneity, i.e., principles to reproduce a result consistently in time and space, or from the perspective of different observers.
- Sensitivity: probability of a positive test result if the subject tested presents the condition.
- Specificity: probability of a negative test result if the subject tested does not present the condition.
- Positive Predictive Value (PPV): defined as the probability of true-positives among all individuals with positive test results.
- Negative Predictive Value (NPV): defined as the probability of true-negatives among all individuals with negative test results.

Studies relating to diagnostic accuracy will be selected using the population, index test, reference test, and diagnosis of interest (PIRD) criteria. The PIRD format is recommended for structuring the inclusion criteria of a systematic review of diagnostic test accuracy [45]. Among frail older adults aged ≥60 years (P), what is the diagnostic accuracy of current frailty screening and frailty-oriented risk-stratification tools (I) at identifying frailty and predicting adverse health outcomes (D) versus comprehensive geriatric assessment (R) at time of admission? Short screening tools, requiring no more than 30 min to administer [46] used to identify frailty, even if not originally designed for this purpose, are of specific interest in this systematic review. Only non-disease specific instruments assessing undifferentiated older adults will be included (e.g., instruments to stratify cardiovascular events or cognitive screening instruments will be excluded). Included studies must describe the derivation of an instrument with at least one validation study available, either internal or external. The PIRD criteria, and those used for studies presenting the clinimetric properties of instruments used in this systematic review, are summarized in Table 1. A tentative list of potentially suitable instruments is included in Table 2 below, based on existing publications.

For the purposes of this study protocol, the reference test for frailty is any CGA performed by trained assessors, as well as measures of established frailty models (i.e., physical phenotype or the deficit accumulation model), provided these are used as part of broader assessment. Most EDs have a triage system in place to prioritize patients based on the acuity of their illness, which, in turn, determines waiting time. Few of these triage systems evaluate the age-specific conditions of older patients on admission to the ED [47]. Rapid administration is required, given the need to assess and stratify patients quickly at or just after triage. Although no specific definition of what constitutes a “short” administration time for a frailty-oriented risk-stratification tools in urgent care settings is available, studies examining screens in other settings have defined “short” as those with 5–14 items, taking between 2 and 5 min to complete [46]. This study will exclude instruments taking more 30 min to apply, and, within the included tools, aims to conduct a sub-analysis comparing the performance (both diagnostic and predictive accuracy) of “short” (2–5 min and 5–14 items), “ultra-short” (with 1–4 items taking <2 min to complete), and “standard” (15 or more items taking >5, but ≤30 min). Instruments where the time taken is not known will be excluded from this sub-analysis. As no single unifying consensus exists on an accepted definition of frailty [4], any recognized, previously published definition will be accepted based on the premise that frailty represents a state of increased vulnerability to minor stressors that results in increased risk of adverse health outcomes [48,49], and based on work by Sezgin et al. [50]. Frailty is a dynamic spectrum with identifiable transitions from non-frail to pre-frail and frail states over time, in either direction [50]. Widely used in the literature, a standardized definition of pre-frailty is also lacking, though it is increasingly being characterized as a very early or mild form of frailty [51,52]. Given this, we will include pre-frailty in the search terms, and attempt to conduct, as a sub-analysis, an examination of the accuracy of available tools for pre-frailty. However, as few short tools are available to measure pre-frailty [53], this may not be possible.

Studies reporting diagnostic accuracy, and validation studies who report predictive accuracy (predominantly observational studies, including case control, longitudinal, and cohort studies), will be included, where screening tools to identify frailty, or other risk stratification tools, are used to identify older adults, and compared to one or more of these reference standards to explore the diagnostic and predictive accuracy of the tool. Case reports, case series, commentaries, opinion papers, conference abstracts, editorials, study protocols, and review articles will be excluded.

In terms of establishing the predictive accuracy of the screening tools, the future event criteria will include adverse short- and long-term outcomes experienced by older people after screening in EDs. Adverse outcomes will specifically include functional decline (change in activities of daily living from a defined baseline using a validated functional scale), prolonged length of stay (LOS) if admitted to hospital (provided defined average LOS is available), acute care utilization within 90 days (unplanned ED representation/re-attendance, unscheduled hospital admission), transfer to long-term care (institutionalization), and falls and mortality within one year. Our study will include only published literature.

### 2.3. Exclusion Criteria

Studies will be excluded if their population mean or median age is <60 years, and where data cannot be extracted separately on those ≥60 years. Full text articles must be available to be included. Full text articles, in our study protocol, refers to papers published in peer reviewed journals, excluding abstract-only (i.e., conference) publications. Studies in which the frailty tool is used in a validation study of another instrument, and studies that report on frailty, but without the use of a clearly identifiable tool, will also be excluded. Papers published before 2000 will not be included as this predates standardized definitions of frailty, i.e., Fried’s frailty phenotype or the deficit accumulation theory. This is in line with previous published studies, including Apostolo et al. [31].

### 2.4. Information Sources/Search Strategy

The search will include studies published from 1 January 2000 to 21 March 2021. The following electronic databases will be searched: PubMed, Bethseda, USA; Cochrane Library, London, United Kingdom; CINAHL, Glendale, USA; Embase, (Elsevier), Amsterdam, Netherlands; TRIP, Newport, United Kingdom and Google Scholar, Mountain View, USA. No language restrictions will be applied. Those published in languages other than English or that of the co-investigators will be translated using Google Translate, Mountain View, USA. The planned search strategy is presented in Table 3, and will be modified if necessary for each database searched. Terms of medical subjects (MeSH) and keywords will be used individually or in combination during the query. The following search terms will be applied: (frail* OR prefrail* OR pre frail*) AND (tool* OR screen* OR scale* OR score* OR measure* OR index* OR instrument* OR prediction*) AND (“emergency department” OR “emergency services” OR hospital*). Studies in all languages will be included and translated by team members fluent in that language or where required, using Google Translate. A provisional natural language search will also be included, with search terms “frailty tools” OR “older adults” OR “emergency department”. The reference lists of all potential publications, including relevant systematic reviews, will be manually retrieved and reviewed to further locate additional frailty screening tools, including Google Scholar and grey literature. Finally, Google Scholar will be searched using the search string: “allintitle: (screen or screening) OR (frail OR frailty)” (63 results). All searches will be imported into the Endnote reference management system, and duplicates will be removed.

### 2.5. Study Selection and Data Extraction

Titles and abstracts will be independently screened for relevance based upon the above inclusion and exclusion criteria by two reviewers. Studies deemed eligible will be read fully, and their suitability for inclusion will be independently determined. Any disagreements will be managed by discussion and consensus agreement. Data will be extracted from the included studies by two independent reviewers using standardized forms that will include data on:

- Study information: author, title, citation, type of publication, journal, country.
- Characteristics of the study: design, objective(s), sample size, study population characteristics, setting, findings, prevalence of frailty in the population.
- General characteristics of the tool(s) used to measure frailty: quantity of domains/items, type of items and their category, mode of administration, administration time, development strategy of screening tool.
- Details of the “gold standard” that the frailty-oriented tool was compared with to examine its diagnostic accuracy, for example, CGA or different models of frailty (deficit accumulation or phenotype).
- Outcome(s) assessed to examine predictive validity, including mortality, hospitalization, and institutionalization, including their time frame.
- Measurement properties of the tools indicated by the COSMIN criteria.
- Other relevant data.

Any disagreement in data extraction will be resolved by discussion. If disagreements persist, a third author will independently extract the data. If a study presents missing, unclear, or incompletely reported data, we will attempt to contact the study authors to obtain the data. If no reply is received from the authors, or missing data cannot be supplied, then, for the meta-analysis, missing data with replacement values will be imputed, treating these as if they were observed. The extent of missing data will be documented in the extraction form.

### 2.6. Risk of Bias and Analysis

The methodological quality of the selected studies will be evaluated independently by two reviewers using the Quality Assessment of Diagnostic Accuracy Studies-Comparative (QUADAS-C) tool, an extension of the validated QUADAS-2 for assessing the quality of diagnostic and prognostic accuracy studies [54]. The QUADAS-C provides an important update for researchers to assess high-quality diagnostic accuracy studies. Like with QUADAS-2, it consists of four key domains, covering patient selection, index test, reference standard, and flow of patients through the study and timing of the index test and reference standard, but, unlike QUADAS-2, is designed for the assessment of test comparisons. The tool is completed in four phases: (1) review question statement; (2) development of review-specific guidance; (3) construct a flow diagram for the primary study; (4) judgement of bias and applicability and includes a risk-of-bias judgment for the accuracy of each test [54]. For the evaluation of prognostic accuracy studies, the Prediction model Risk of Bias Assessment Tool (PROBAST) will be used [55]. This includes 20 questions across four domains: participants, predictors, outcome, and analysis. Disagreements will be resolved by a third reviewer.

### 2.7. Data Synthesis and Analysis

The studies selected may differ considerably in design, methods, and outcome measures. The initial strategy for data analysis will be the use of descriptive statistics. Summary estimates of sensitivity and specificity with 95% confidence intervals (CIs) will be calculated using the bivariate random effects model. Meta-analysis will be considered if the appropriate conditions are met in certain subgroups. If data are available, a subgroup analysis will be conducted according to variations in the characteristics of the trial participants and frailty screening tools. When considerable heterogeneity is detected in an analysis, a subgroup analysis will be performed if necessary [56]. Sensitivity analysis will be conducted to monitor the robustness of primary decisions made in the review process. Certain decisions, such as sample size, methodological weakness, and missing data, will be considered. The results of the sensitivity analysis will be presented in summary tables. Sub-analysis will be conducted based on age, sex, type of instrument (frailty-specific and frailty-oriented risk-prediction instrument), and administration time as described above. Bias risk in the review process, as indicated by the results of the sensitivity analysis, will be discussed. Quantitative data will, where possible, be pooled in statistical meta-analysis using Rev Man software (Review Manager [Computer Program] Version 5.4. The Cochrane Collection 2020] to perform data synthesis. The significance threshold will be *p* < 0.05 on two sides. A forest plot for each parameter will be constructed to indicate the weight ratio of each incorporated study. Two models of meta-analysis will conduct for outcomes: the fixed-effect model and the random-effect model [57].

Heterogeneity will be assessed by the Higgins’ *I*^2^ statistical test [58]. The following cut-offs for the degree of heterogeneity will be applied: *I*^2^ = 0–40%: might not be important; *I*^2^ = 30–60%: may represent moderate heterogeneity; *I*^2^ = 50–90%: may represent substantial heterogeneity; *I*^2^ = 75–100%: considerable heterogeneity. The random-effect model is more appropriate when heterogeneity is present, where the *I*^2^ test is between 50 and 75%. If the *I*^2^ test is higher than 75%, we will find the possible reasons from both clinical and methodological perspectives, and provide an explanation or conduct subgroup analysis. Where statistical pooling is not possible, the findings will be presented in narrative form, including tables and figures to aid in data presentation where appropriate. These figures will comprise graphic flowcharts to illustrate the statistical methods used.

## 3. Discussion

Frailty is a dynamic and potentially reversible condition that contributes to functional decline in older adults [59]. Understanding an individual’s frailty status at admission to hospital can help predict adverse outcomes [60]. Early identification and management of frailty improves care [61], facilitating the introduction of tailored pathways and interventions to reduce the risk of a range of adverse healthcare outcomes [62]. This can improve the efficiency of acute hospital services by allowing the cohorting of appropriate patients to older persons’ or frailty-specific units [17,63], by promoting early allocation of CGA, which is known to reduce mortality and institutionalization [61]. It also facilitates redirection to more appropriate care in the community, such as hospital at home or day hospital services [19,48]. Further, risk stratification is necessary because CGA is a limited resource and not every patient will require it [64]. Given these points, it is important to identify frailty promptly, as early into an admission as possible. Reflecting this, many authorities, such as the British Geriatrics Society, recommend that all encounters between health and social care staff and older people include an evaluation of frailty [65].

Rapid geriatric screening should be adaptable to the ED environment. This is borne out of both practicality and necessity. Traditionally, the ED was designed to cater for trauma and acute critical illness. This model is not conducive to catering for the complex care needs of frail older patients [66,67,68]. Frailty pathways and rapidly administered screening instruments validated for use in the ED may assist in targeted frailty interventions [69,70]. Medical educators and policy makers advocate for focused, evidence-based screening efforts to optimize geriatric outcomes [38]. Thirty years ago, the landmark Society for Academic Emergency Medicine (SEAM) taskforce completed its work on the care of older patients in EDs [68]. As a result of the taskforce recommendations, geriatric focused EDs have been developed and implemented in the United States to respond to the unique needs of older adults [69]. Such improvements range from the training of specialist geriatric medicine staff to the modification of environments, including the use of “quiet areas” and ambient lighting. These geriatric EDs specifically address the unique medical, physical, social, and psychological needs of older adults [70]. As the number of geriatric EDs continues to expand, so will the demand to derive and validate more accurate risk stratification instruments. Irrespective, there will remain a need to identify frailty among unselected older people presenting to EDs.

Insufficient data on the nature of screening instruments have prompted geriatric and emergency medicine researchers to list development and implementation trials of prognostic screening instruments among the highest research priorities in this field [71,72]. The ideal frailty screening instrument for use in EDs should be well-calibrated across a broad range of illness severity, disability, and socioeconomic strata [73]. The most recent review included data published before 2013 and found that insufficient data on the clinimetric properties of such instruments in EDs were available at the time [73]. Our preliminary search suggests that many papers have been published since 2012, in keeping with the growing recognition that the early identification of frailty can promote better care in EDs [22]. This systematic review and meta-analysis will therefore provide important up-to-date information about the quantity and quality of studies regarding the diagnostic and predictive accuracy of frailty screening instruments for older adults attending EDs to support practice. Our study protocol differs from existing publications as it focuses on the emergency department environment, an area that has a paucity of previous published protocols. The two most recent frailty assessment study protocols did not include the ED as a study clinical setting [38,39]. Our protocol will therefore add much needed data on the acute care frailty environment and aligns with the new EUGMS GEMS guidelines to develop age/frailty risk stratification models through use of frailty screening tools in EDs [21].

The on-going COVID-19 pandemic has shown that this is timely by highlighting the importance of the early recognition of frailty as a way to improve the triage of older patients and identify those most likely to benefit from critical and intensive care [74]. It has also highlighted that identifying frailty in acute care settings through the utilization of screening instruments by those without geriatric training is challenging. Appropriate instruments should be used, and training is required. Without adequate knowledge of frailty-oriented risk-stratification tools, there is concern that decisions regarding the prioritization and escalation of care could be made based on age or initial impressions, rather than on the need, or more importantly, the potential to benefit [74,75]. In those with acute illness, a detailed collateral and understanding of a patient’s baseline function rather than their current status should be obtained, to more accurately determine frailty status [76]. Education of clinical staff based on a better understanding of the features of validated frailty screening tools in the ED is a prerequisite [77], particularly as a limited number of study protocols have been published on frailty assessment, often focusing on surgical trauma [78] or community settings [38].

Understanding which instruments are most accurate, reliable, and quick to administer will help support the training of ED staff.

### 3.1. Limitations

Some potential limitations based on previous systematic reviews include the potential for statistical heterogeneity, even when assessing the same instrument for the same outcomes on similar patient groups. This is partially due to inconsistent definitions for outcomes, and variable methods for measuring outcomes [36]. Given the lack of a single consensus definition of frailty [4], this is a significant potential risk in this review. In addition, some published studies have enrolled patients exclusively from EDs, whereas others have included patients recruited after ED admission. A further potential issue may be missing data, limiting the scope of the statistical analysis. Several confounding variables particular to an older population, such as cognitive impairment and lack of access to primary care due to factors such as immobility, may limit bias estimates of prognostic accuracy of the screening instruments. Lastly, limiting the systematic review to five databases may result in some relevant papers being missed.

### 3.2. Dissemination

The review will be published in a peer-reviewed journal and presented at appropriate national and international conferences.

## 4. Conclusions

This protocol describes the plan for a comprehensive, up-to-date systematic review and meta-analysis exploring the diagnostic accuracy for frailty and predictive validity for a range of healthcare outcomes for all available frailty screens and frailty-oriented risk prediction instruments for use with older people in EDs, exploring the clinimetric properties of available instruments. Analysis and application of the findings of this in real-world settings will yield meaningful results to support risk-stratification, education, and training for staff working with vulnerable older adults presenting acutely unwell to EDs.

## Figures and Tables

**Table 1:** Question format criteria for the review strategy, including the population, index test, reference test, diagnosis of interest (PIRD) format for diagnostic accuracy studies, and COnsensus-based Standards for Health Measurement INstruments (COSMIN) for studies examining the clinimetric properties of instruments.

PIRD Question Format			
Population	Index Test	Reference Test	Diagnosis of Interest
Adults aged ≥60 years attending ED using any recognized definition of frailty	Short Screening and risk-stratification tools used to identify frail adults	Comprehensive Geriatric Assessment or measures of established frailty models provided as part of an independent patient review	Accurate identification of frailty and prediction of selected adverse health outcomes
**COSMIN Question Format**			
**Name of Instrument**	**Population**	**Type**	**Measurement Properties**
-	Adults aged ≥60 years attending ED using any recognized definition of frailty	Short Screening and risk-stratification tools used to identify frail adults	Reliability, sensitivity, specificity, validity, positive and negative predictive value

**Table 2:** Provisional list of frailty screening tools validated for use in emergency departments (ED).

Instrument	Author	Year	Age Group (Years)	Administration Time (N/A = Not Available)
Triage Risk Screening Tool (TRST)	Pfiffer et al.	2020	≥75	N/A
Hospital Frailty Risk Score (HFRS)	Gilbert et al.	2018	≥75	N/A
Brief Risk Identification for Geriatric Health Tool (BRIGHT)	Boyd et al.	2008	≥75	N/A
International Resident Assessment Instrument (Inter RAI) ED- Screen	Costa et al.	2017	≥70	1 min
Short Emergency Geriatric Assessment (SEGA)-French	Schoevaerdts et al.	2004	≥70	10mins
Criteria for Screening and Triaging to Appropriate Alternative Care (CRISTAL)	Cardona et al.	2018	≥65	<5 min
Identification of Seniors at Risk (ISAR)	Salvi et al.	2012	≥65	N/A
Clinical Frailty Scale (CFS)	Rockwood et al.	2005	≥65	5 min

**Table 3:** Search strategy with number of citations predicted according to each database.

Search Details	PubMed	CINAHL	Cochrane	Embase	Google Scholar	TRIP	TOTAL
Search #1 FRAIL * OR PREFRAIL * OR PRE FRAIL*	18,514	8857	3967	24,609	N/A	11,797	43,135
Search #2 TOOL * OR SCREEN * OR SCALE * OR SCORE * OR INSTRUMENT * OR MEASURE * OR INDEX * OR RISK OR PREDICTION *	949,076	376,975	1,529,902	1,400,342	N/A	1,801,459	6,057,754
Search #3 “EMERGENCY DEPARTMENT” OR “EMERGENCY SERVICES” OR HOSPITAL *	929,624	305,742	309,120	1,272,570	N/A	96,115	2,913,171
#1 AND #2	10,199	3008	3967	18,819	N/A	60,259	79,322
#1 AND #3	8056	3008	1488	13,605	N/A	1427	27,584
#1 AND # 2 AND #3	4972	3008	1480	10,684	63	1281	21,488
Predicted citation count							21,488

* Refers to truncation; a symbol added to the end of the root of a word to instruct the database to search for all forms of a word. “” refers to quotation marks; a symbol that instructs the database to search for an exact phrase.

## Data Availability

Not applicable.
